# RTA‐408 protects against propofol‐induced cognitive impairment in neonatal mice via the activation of Nrf2 and the inhibition of NF‐κB p65 nuclear translocation

**DOI:** 10.1002/brb3.1918

**Published:** 2020-12-09

**Authors:** Ling Zhang, Qian Zhou, Chun‐Li Zhou

**Affiliations:** ^1^ Department of Anesthesiology Jingzhou Central Hospital, The Second Clinical Medical College, Yangtze University Jingzhou China; ^2^ Department of Anesthesiology Xiangyang Central Hospital Affiliated Hospital of Hubei University of Arts and Science Xiangyang China

**Keywords:** cognitive dysfunction, NF‐κB, Nrf2, propofol, RTA‐408

## Abstract

**Objective:**

To explore the effect of RTA‐408 on the propofol‐induced cognitive impairment of neonatal mice via regulating Nrf2 and NF‐κB p65 nuclear translocation.

**Methods:**

C57BL/6 neonatal mice were randomized into intralipid, propofol, vehicle + propofol, and RTA‐408 + propofol groups. The learning and memory ability was inspected by Morries water maze (MWM) test. TUNEL staining was performed to examine the apoptosis of neurons in hippocampus. The gene and protein expressions in hippocampus were detected by immunohistochemistry, qRT‐PCR, or Western blotting. The activities of glutathione peroxidase (GPx), superoxide dismutase (SOD), and catalase (CAT) were tested by the corresponding kits.

**Results:**

Propofol prolonged escape latency of mice, decreased the times of crossing the platform, and shortened the time of staying in the target quadrant, while RTA‐408 treatment improved the above‐mentioned situation. Besides, Nrf2 protein in hippocampus of mice induced by propofol was decreased with the increased NF‐κB p65 nuclear translocation, which was reversed by RTA‐408. Meanwhile, RTA‐408 decreased the apoptosis of neurons accompanying with the down‐regulation of Caspase‐3 and the up‐regulations of neuronal‐specific nuclear protein (NeuN), microtubule‐associated protein 2 (Map2), Ca_2_^+^/Calmodulin‐dependent Protein Kinase II (CaMKII), and parvalbumin (PV) immunostaining in hippocampus. Besides, propofol‐induced high levels of proinflammatory cytokines and antioxidase activities in hippocampus were reduced by RTA‐408.

**Conclusion:**

RTA‐408 improved propofol‐induced cognitive impairment in neonatal mice via enhancing survival of neurons, reducing the apoptosis of hippocampal neurons, mitigating the inflammation and oxidative stress, which may be correlated with the activation of Nrf2 and the inhibition of NF‐κB p65 nuclear translocation.

## Graphical abstract

Nrf2 activator RTA‐408 can be used to reduce the apoptosis of hippocampal neurons, lower the inflammatory response and oxidative stress, and improve learning and memory capacity by restricting propofol‐induced NF‐κB nuclear translocation of hippocampus of neonatal mice.

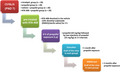

## 1 INTRODUCTION

A series of animal studies have demonstrated that many anesthetics, like ketamine, propofol, etomidate, and isoflurane, could cause damage to the brain neurons during the early postnatal period, further leading to cognitive and neurological abnormalities (Kletecka et al., 2019). Since propofol (2, 6‐diisopropylphenol) has rapid onset and short duration of action, it has been widely used in surgical procedure and intensive care units (ICU), as well as the pediatric ICU (Yan et al., 2019), which, however, could trigger the apoptosis or inhibit the neuronal growth in vitro (Wang et al., 2018). Especially, the long‐term repeated injection of large‐dose propofol has influence on the learning and memory capacity (Xu et al., 2016), which could be used as the regulator of Nrf2 and nuclear factor‐κB (NF‐κB) pathways (Jiang et al., 2018).

Nrf2 is a basic leucine zipper transcription factor, belonging to the Cap' n' Collar (CNC) transcription factor family, which was functioned as a key transcription factor and a major regulator of cell oxidative stress, inducing the expression of antioxidant enzymes, detoxification enzymes, and downstream protein molecules (Ikram et al., 2019). Currently, the researches of Nrf2 in the central nervous system have become a hot spot (Liu et al., 2019; Shah et al., 2019). NF‐κB is a family of dimeric transcription factors made up of five family members, p50 (NF‐κB1), RelA (p65), p52 (NF‐κB2), c‐Rel, and RelB (Oda‐Kawashima et al., 2020). Zhong et al. (2014) revealed down‐regulation of p65 was involved in the potential mechanisms of propofol‐induced neurotoxicity. RTA‐408 (Omaveloxolone), a nuclear factor E2 related 2 (Nrf2) activator, is a synthetic triterpeniod with anticancer and anti‐inflammatory activities (Probst et al., 2015). Recently, Yang et al. (2019) demonstrated RTA‐408 attenuated IL‐1β‐stimulated p65 nuclear translocation in a rat brain astrocyte (RBA‐1) line. Therefore, we hypothesis that RTA‐408 may play a role in the propofol‐induced cognitive dysfunction in neonatal mice via affecting Nrf2 and NF‐κB p65. In our experiment, C57BL/6 mice aged 7 days were pretreated with RTA‐408, and treated with propofol for 6 hr (Mandyam et al., 2017), to explore the treatment of RTA‐408 on propofol‐induced cognitive dysfunction of neonatal mice.

## 2 MATERIALS AND METHODS

### 2.1 Ethical statement

All experiments conducted on animals in the study are in accordance with the management and using principles of local experimental animals, meet the requirements of animal ethics, and follow the *Guidelines for the Management and Use of Experimental Animals* issued by the National Institutes of Health of the United States (Mason & Matthews, 2012).

### 2.2 Anesthesia

**FIGURE 1:**
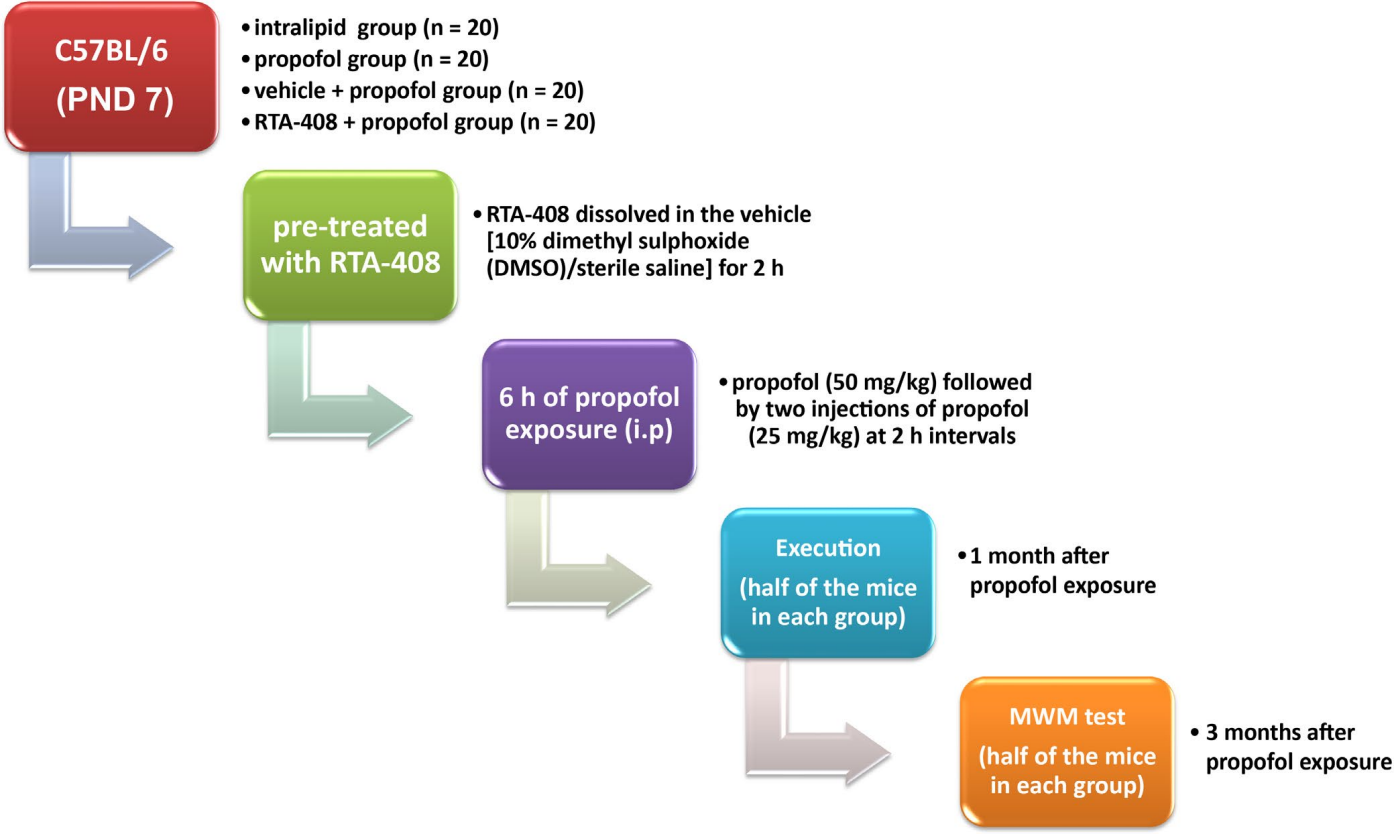
Brief flow chart of the experimental procedures

C57BL/6 wild‐type male mice aged 7 days (PND 7, purchased from Jackson Laboratory) were fed in clean animal rooms with normal circadian rhythm and free eating and drinking at room temperature 22°C. Mice were randomly divided into four groups: intralipid group, propofol group, vehicle + propofol group, and RTA‐408 + propofol group. The animals grouping and the experiment process were shown in Figure 1. The mice in the vehicle + propofol or RTA‐408 + propofol were pretreated with either vehicle [10% dimethyl sulphoxide (DMSO)/sterile saline] or RTA‐408 (Selleck Chemicals) for 2 hr (Shekh‐Ahmad et al., 2018). Then, the mice in intralipid group and propofol groups received intraperitoneal (i.p.) injection of intralipid (vehicle control, 50 mg/kg; Cutter Laboratories) and propofol (50 mg/kg; Sigma‐Aldrich) followed by twice injections (25 mg/kg, i.p.) of intralipid and propofol at 2‐hr intervals, respectively, 6 hr of exposure in all (Pearn et al., 2018). One month after propofol exposure, half of the mice randomly chosen in each group were killed, and the others were fed for 3 months after propofol exposure for Morris Water Maze (MWM) test. The body weight was recorded every week.

### 2.3 Sample collection

The mice in each group were weighted and then killed after blood samples collected from their tail vein. The chest cavity was quickly opened to fully expose the heart. A 16G vein puncture needle was used to insert into the aorta through the left ventrical, and the right auricle cut to be quickly infused with 200 ml of ice normal saline. The brain was taken out, quickly transferred into the ice block formed by sterile double steaming water, and hippocampus, was separated, washed with 4°C sterile water and dried to be frozen in liquid nitrogen, and then transferred to keep it in refrigerator at −80°C. Corresponding kits were used to test the activities of glutathione peroxidase (GPx), superoxide dismutase (SOD), and catalase (CAT). The hippocampus was perfused rapidly with heparinized normal saline and fixed with 200 ml of 4% paraformaldehyde.

### 2.4 qRT‐PCR

TRIzol reagent was used to extract the total RNA, which was then reversed into cDNA by the Super Script III cDNA synthesis kit (Bio‐Rad Laboratories, Inc., Hercules, CA, USA). A SYBR Green PCR Master kit with an ABI Prism 7,500 Sequence Detection System (Applied Biosystems, Foster City, CA) was conducted to perform the PCR reaction. Table 1 shows primer sequences used in the study. The 2^−△△Ct^ method was utilized to calculate the relative expression of target gene with β‐actin as the internal reference.

**TABLE 1:** Primer sequences of qRT‐PCR in the study

Gene	Primer sequences
IL‐6	Forward: 5′‐ATGAAGTTCCTCTCTGCAAGAGACT‐3′ Reverse: 5′‐CACTAGGTTTGCCGAGTAGATCTC‐3′
IL‐1β	Forward: 5′‐GAGTGTGGATCCCAAGCAAT‐3′ Reverse: 5′‐AGACAGGCTTGTGCTCTGCT‐3′
TNF‐α	Forward: 5′‐CAAAGGGAGAGTGGTCAGGT‐3′ Reverse: 5′‐ATTGCACCTCAGGGAAGAGT‐3′
β‐actin	Forward: 5′‐GGTCATCACTATTGGCAACG‐3′ Reverse: 5′‐ACGGATGTCAACGTCACACT‐3′

### 2.5 Western blotting

The hippocampus was dispersed in precooled RIPA lysis buffer, followed by homogenization. Nuclear and cytoplasmic proteins were extracted using NE‐PER Nuclear and Cytoplasmic Extraction Reagents (Pierce Biotechnology, Inc.) followed by the quantitation with a bicinchoninic acid (BCA) protein assay kit (Thermo Scientific) according to the instructions. The same sample was separated by sodium dodecyl sulfate–polyacrylamide gel electrophoresis (SDS‐PAGE), transferred to PVDF membrane. 5% nonfat milk was added for 1 hr followed by the addition of primary antibodies overnight at 4°C. After the membrane was washed once for three times, secondary antibody was added, and the membrane was washed three times after incubation at 37°C for 1.5 hr (each time for 10 min), followed by the detection with ECL (Amersham Pharmacia Biotech). The densitometric analysis was conducted using ImageJ Software.

### 2.6 TUNEL staining

The hippocampus was dehydrated with gradient ethanol, cleared in xylene, embedded in paraffin, and sectioned into 4 μm slices. Then, these sections were subjected to conventional dewaxing to water. The TUNEL kits (Boehringer Mannheim) were applied to conduct TUNEL staining according to the instructions. Observed under the optical microscope, the nucleus of apoptotic cells was stained into brown or yellow. The percentage of TUNEL positive cell number was calculated in five arbitrarily selected fields.

### 2.7 Immunohistochemistry

Sections were dewaxed, incubated with 3% H_2_O_2_ at room temperature for 5–10 min, then washed with PBS, and incubated for 10 min at room temperature. The Caspase‐3 (1/100, Abcam, USA), NeuN (1:1,000; Chemicon), PV (1:2,000; Sigma‐Aldrich), NF‐κB p65 (1:200; Santa Cruz Biotechnology), Map2 (1/8,000; Abcam), and CaMKII (1:500; Abcam) were added into it at 4°C overnight. After being washed with PBS, the secondary antibody was added and incubated at 37°C for 10–30 min, which was followed by the addition with alkaline phosphatase‐labeled streptavidin at 37°C for 10–30 min. Then, the tissues were washed with PBS (5 min × 3 times), developed with DAB, counterstained with hematoxylin, dehydrated, rendered transparent, and mounted.

### 2.8 Morris water maze test

The device adopted a diameter of 214 cm with a height of 50 cm and a depth of 30 cm, and four white mark points divided the pool into four quadrants. Mice were put into the pool from different quadrants every time, and the time from entering water to finding platform was calculated as the escape latency. Mice, which cannot find the platform in 60 s, were placed on the platform to 10 s. After 5 days of continuous training, 4 times a day, each time from different quadrants with different random entry points, the average value of daily test was taken as the learning and memory performance. The platform was removed in the morning after the end of the above experiment. The mice entered the water in the opposite of the original quadrant, and the time of staying in the target quadrant was recorded.

### 2.9 Statistical analysis

SPSS 22.0 statistical software was used for conducting analysis. The measurement was expressed in the way of mean ± standard deviation (*SD*). One‐way or two‐way ANOVA was used as a tool for comparison among multiple groups followed by Tukey's honestly significant differences (HSD) test or Holm‐Sidak method for pair comparison, respectively. *p* < .05 was taken as statistically significant.

## 3 RESULTS

### 3.1 RTA‐408 strengthened the learning and memory capacity of propofol‐induced neonatal mice

**FIGURE 2:**
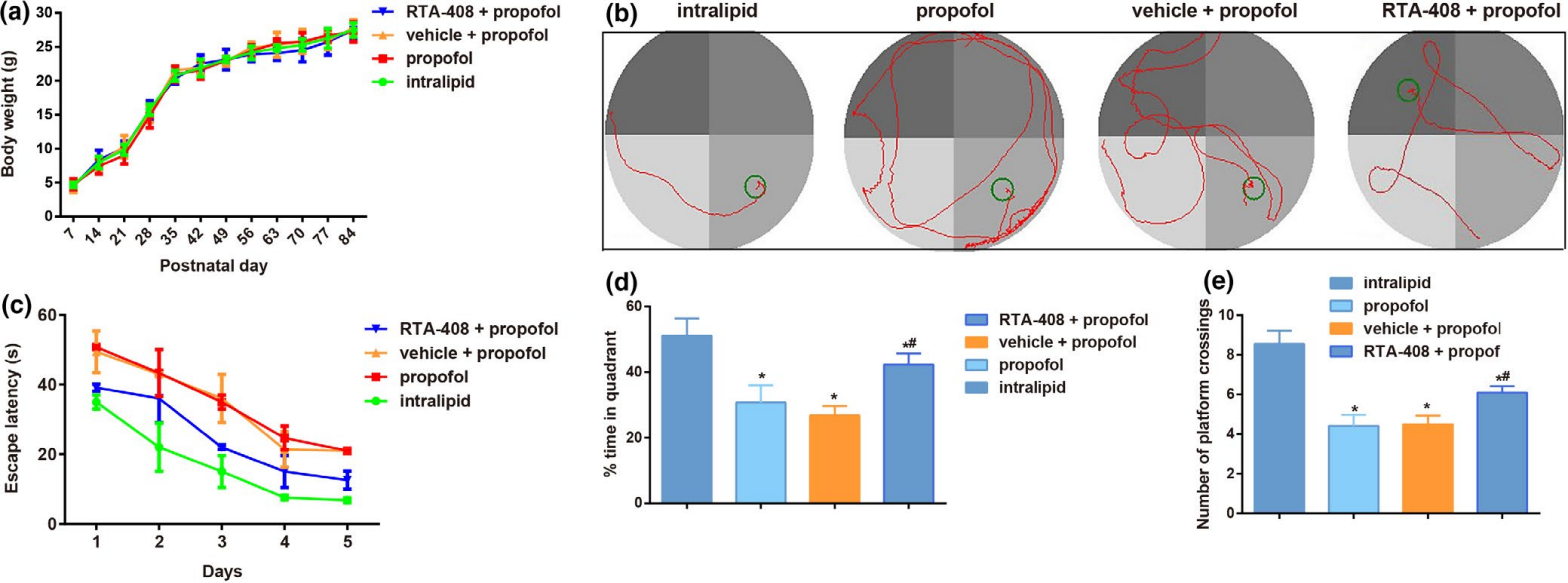
RTA‐408 improved the learning and memory abilities of propofol‐induced mice. Notes: (a) Changes in body weight of mice from PND7 to PND84 (3 months) (two‐way ANOVA followed by pairwise comparison with post hoc Holm‐Sidak method); (b) Representative images of MWM test of the mice in four groups; (c) Escape latency period of mice in each group (two‐way ANOVA followed by pairwise comparison with post hoc Holm‐Sidak method); (d) Percentage time of staying in the target quadrant of mice in each group (One‐way ANOVA followed by Tukey’ HSD test). (e) The number of platform crossings (One‐way ANOVA followed by Tukey' HSD test); Data are expressed as means ± *SD*, and *n* = 10 mice per group; *p < .05, compared with intralipid group, ^#^ *p* < .05, compared with propofol group and vehicle + propofol group

No significant difference was observed in body weight (*p* > .05, Figure 2a) among groups. Morris water maze test showed (Figure 2b–e) that the period of propofol‐induced escape latency was significantly prolonged, with the decreased number of platform crossings (both *p* < .05). By comparison with propofol group, the escape latency of mice in RTA‐408 + propofol group was reduced, but the number of platform crossings was increased (both *p* < .05). Besides, when compared with intralipid group, the time of mice in the target quadrant in propofol group was shortened (*p* < .05), but it was prolonged in RTA‐408 + propofol group, as compared to propofol group.

### 3.2 Comparison of Nrf2 and NF‐κB expression in hippocampus of neonatal mice in each group

**FIGURE 3:**
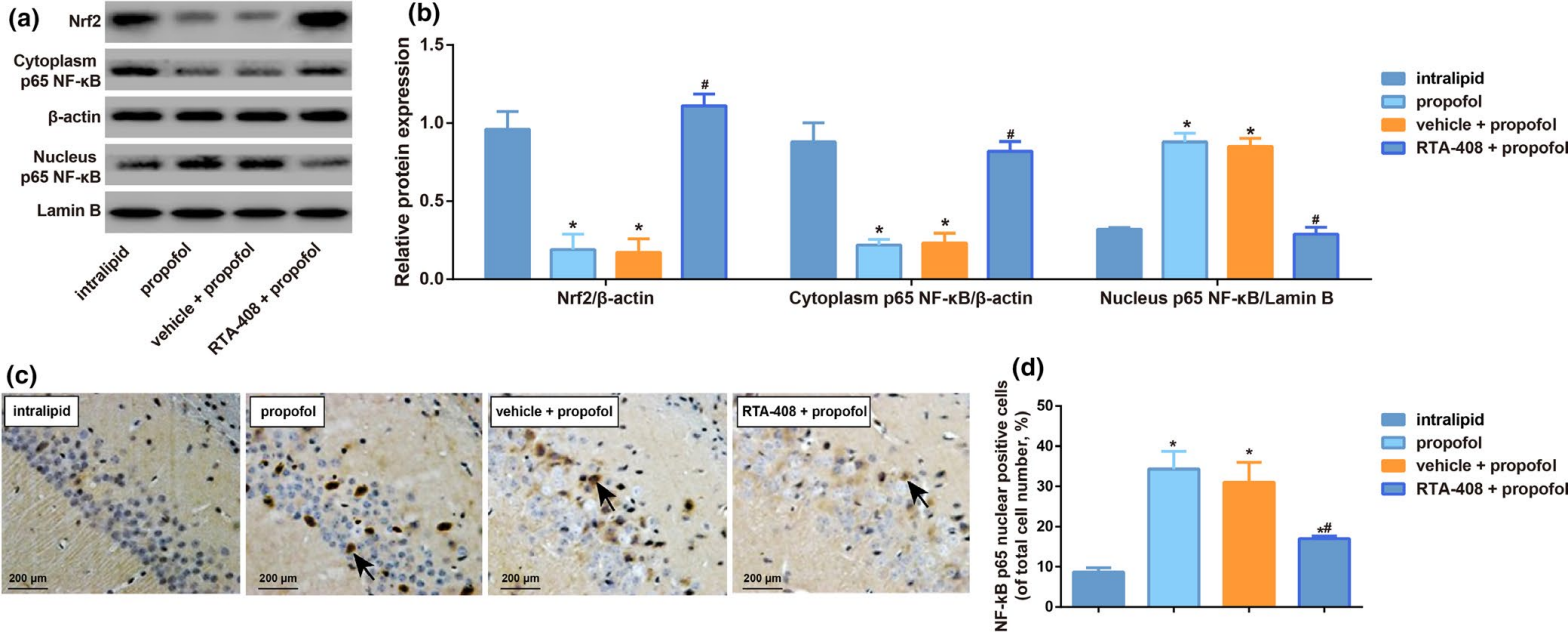
Effect of RTA‐408 on Nrf2 and NF‐κB p65 nuclear translocation in hippocampus from propofol‐induced mice. Note: A‐B: The expression of Nrf2 and NF‐κB p65 nuclear translocation in the hippocampus of mice detected by Western blotting; C: NF‐κB p65‐positive neurons were detected in CA1 subfield cells using immunohistochemical staining. D: Quantitative analysis of NF‐kB p65 nuclear‐positive cells. The data are expressed as percentage of total cell number. Data are expressed as means ± *SD*, and analyzed using One‐way ANOVA followed by the Tukey’ HSD test, *n* = 10 mice per group; * *p* < .05, compared with intralipid group; # *p* < .05, compared with propofol group and vehicle + propofol group

The result of Western blotting demonstrated the decreased Nrf2 protein and increased NF‐κB p65 nuclear translocation in hippocampus of mice from propofol group and vehicle + propofol group as compared with intralipid group (all *p* < .05, Figure 3a,b). Additionally, protein expression of Nrf2 in the hippocampus of mice from RTA‐408 + propofol group was up‐regulated, while NF‐κB p65 nuclear translocation was inhibited as compared with propofol group (all *p* < .05). The NF‐κB p65 nuclear‐positive cells were increased in the propofol group by the immunohistochemistry, which was decreased in the RTA‐408 + propofol group (Figure 3c,d).

### 3.3 RTA‐408 increased the propofol‐induced loss of mature neurons in neonatal mice

**FIGURE 4:**
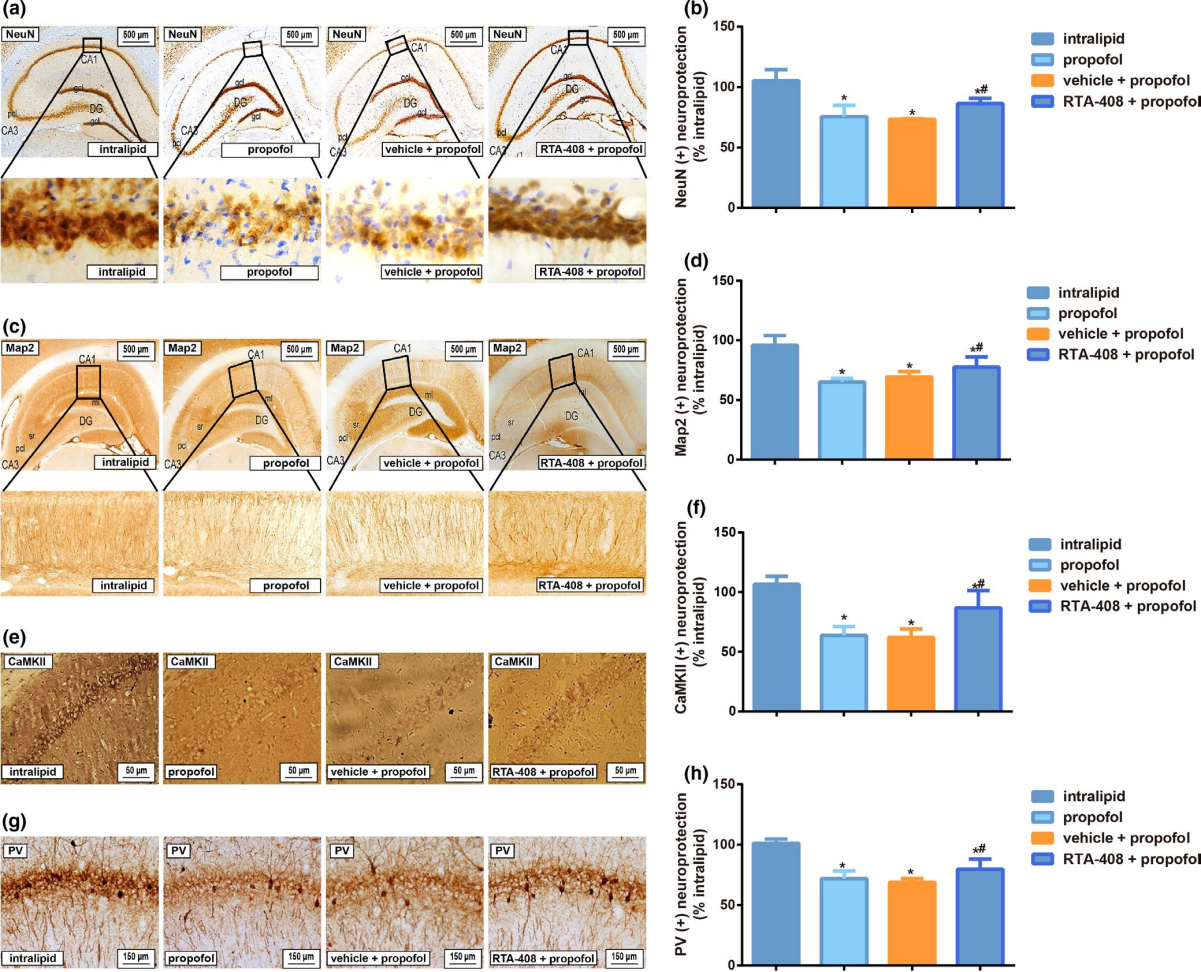
Comparison of NeuN, Map2, CaMKII, and PV immunostaining in the hippocampus from mice among different groups. Note: A‐B: Representative images of the NeuN staining from hippocampus of mice with high magnification of boxed CA1 areas (A), and the percent protection of NeuN (+) cells in various subgroups in the hippocampus (B). C‐D: Representative image of the Map2 staining from hippocampus of mice with high magnification of boxed CA1 areas (C), and the percent protection of Map2 (+) cells in the hippocampus from mice among the various subgroups (D); E‐F: Representative image of the CaMKII staining in the hippocampal CA1 areas of mice (E), and the percent protection of CaMKII (+) cells among the different groups (F); G‐H: Representative image of the PV staining in the hippocampal CA1 areas of mice (G), and the percent protection of PV (+) cells among the different groups; ml, molecular cell layer; DG, dentate gyrus; sr, stratum radiatum; pcl, pyramidal cell layer; gcl, granule cell layer. Data are expressed as means ± *SD*, and analyzed using One‐way ANOVA followed by the Tukey’ HSD test, *n* = 10 mice per group; * *p* < .05, compared with intralipid group, ^#^ *p* < .05, compared with propofol group and vehicle + propofol group

According to the NeuN and Map2 immunostaining in the hippocampus as shown in Figure 4a–d, the NeuN and Map2 expressions were decreased in the hippocampal CA1 areas of mice after propofol induction (*p* < .05), which was not differ from those in the vehicle + propofol group (*p* > .05). However, compared with vehicle + propofol group, the NeuN and Map2 expressions in the hippocampal CA1 areas were increased in propofol‐induced mice after RTA‐408 treatment, but were still less than those in the intralipid group (both *p* < .05).

### 3.4 RTA‐408 increased the propofol‐induced loss of pyramidal neurons and interneurons in neonatal mice

As shown in Figure 4e–h, the pyramidal neurons (marked by CaMKII) and interneurons (marked by PV) was decreased in the hippocampal CA1 areas of mice form the propofol group as compared with the intralipid group (both *p* < .05), which was partly reversed by the RTA‐408 treatment (both *p* < .05).

### 3.5 RTA‐408 restricted the inflammation of hippocampus in propofol‐induced neonatal mice

**FIGURE 5:**
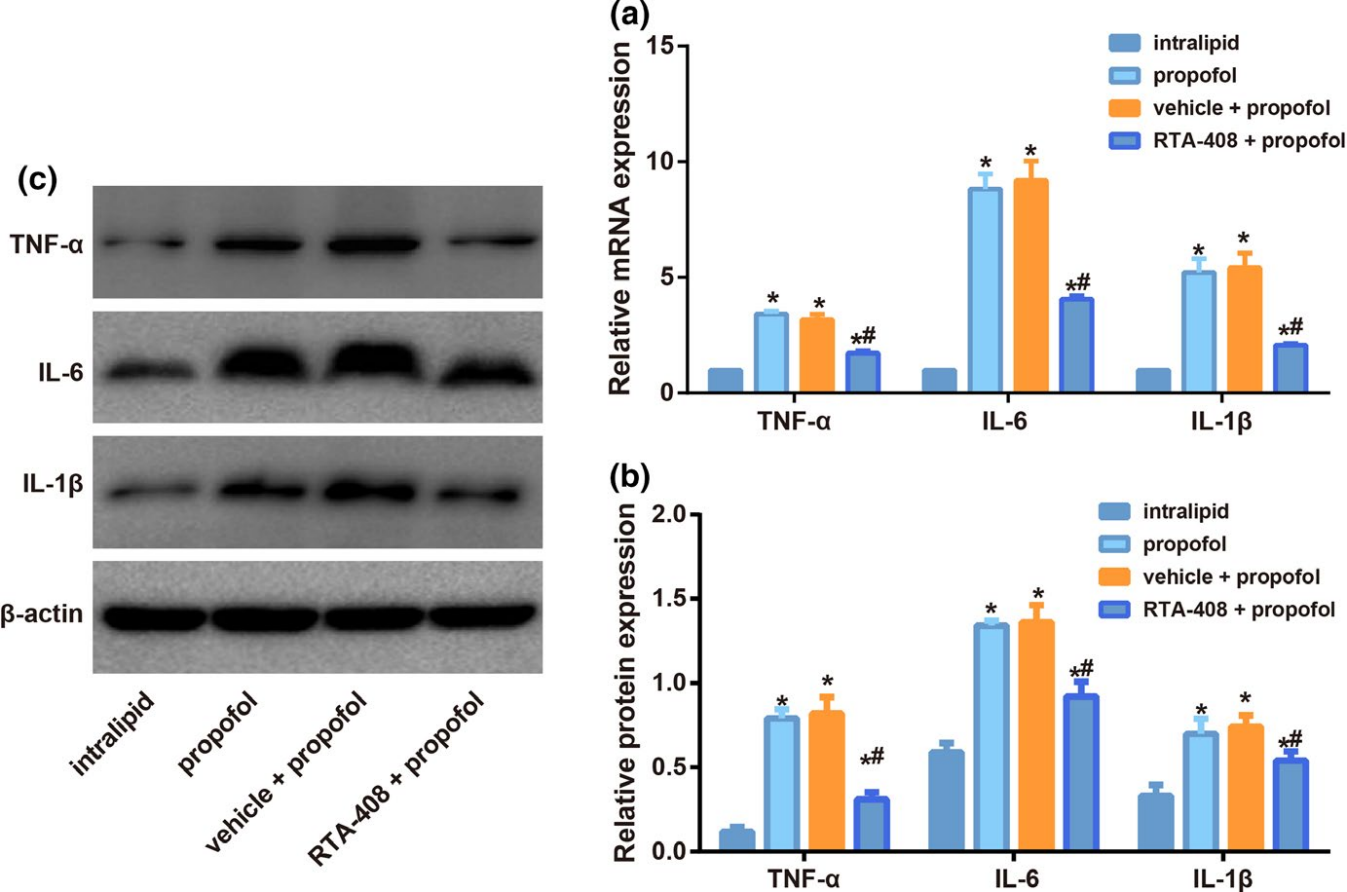
The levels of proinflammatory factors (TNF‐α, IL‐6, and IL‐1β) in hippocampus of mice in each group measured by qRT‐PCR (a) and Western blotting (b–c). Note: Data are expressed as means ± *SD* and analyzed using one‐way ANOVA followed by the Tukey' HSD test, *n* = 10 mice per group;*p < .05, compared with intralipid group, ^#^ *p* < .05, compared with propofol group and vehicle + propofol group

Levels of proinflammatory factors (TNF‐α, IL‐6, and IL‐1β) in the hippocampus of each group were determined by qRT‐PCR and Western blotting in Figure 5, which showed increased levels of proinflammatory factors in the hippocampus of mice from propofol group than those from intralipid group (all *p* < .05), but RTA‐408 pretreatment could decreased the inflammation of hippocampus in propofol‐induced neonatal mice with decreased levels of TNF‐α, IL‐6, and IL‐1β (all *p* < .05).

### 3.6 RTA‐408 reduced propofol‐induced apoptosis and oxidative stress in hippocampus of neonatal mice

**FIGURE 6:**
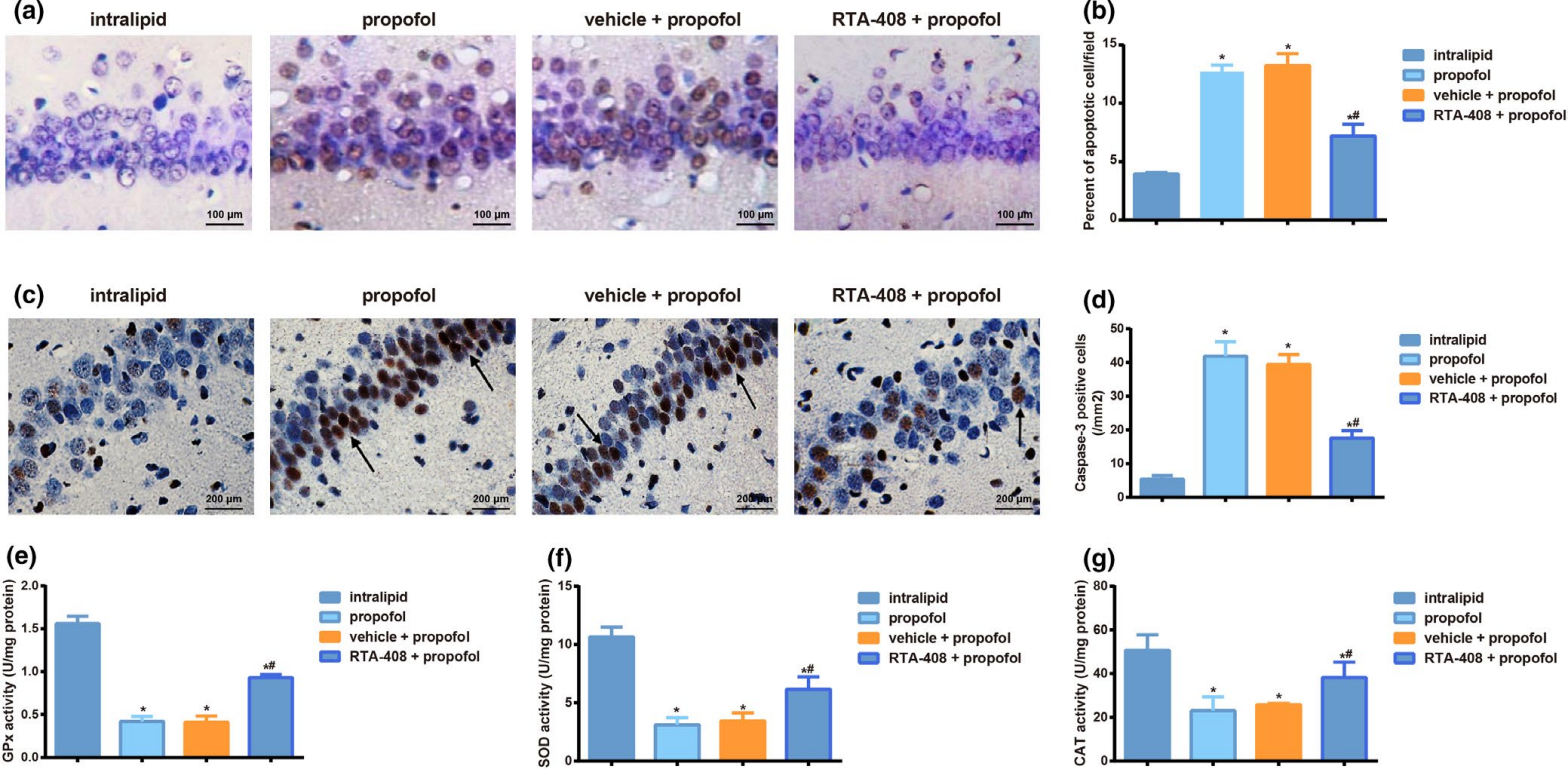
RTA‐408 reduced propofol‐induced apoptosis of hippocampus neurons of neonatal mice and oxidative stress in the hippocampus. Notes: (a, b) TUNEL staining was used to detect apoptosis in the hippocampal CA1 area of mice; (c,d) Immunohistochemistry was to detect the expression of Caspase‐3 in the hippocampal CA1 area of mice; Arrows indicated Caspase‐3 positive staining. (e–g) Comparison of oxidative stress indexes (GPx, SOD, and CAT) in hippocampus of mice; Data are expressed as means ± *SD* and analyzed using One‐way ANOVA followed by the Tukey' HSD test, *n* = 10 mice per group; *p < .05, compared with intralipid group, ^#^ *p* < .05, compared with propofol group and vehicle + propofol group

RTA‐408 pretreatment significantly reduced propofol‐induced apoptosis of hippocampus neurons of mice (*p* < .05, Figure 6a,b). Besides, expression of Caspase‐3 was tested in the CA1 area of hippocampus via immunohistochemistry, which showed Caspase‐3 expression was increased significantly in other three groups compared with intralipid group, but when compared to vehicle + propofol group, its expression was decreased significantly in RTA‐408 + propofol group (all *p* < .05, Figure 6c,d). Moreover, the activities of GPx, SOD, and CAT in the hippocampus of mice from propofol group and vehicle + propofol group were lower than those from intralipid group, while RTA‐408 can significantly improve propofol‐induced oxidative stress in hippocampus (all *p* < .05, Figure 6e–g).

## 4 DISCUSSION

According to the previous study, the brain from a one‐week‐old mouse developed similar as the human's brain to some extent (Pearn et al., 2018). In addition, propofol has been pointed out to cause the growth cone collapse, axonal transport impairment, loss of synaptic connectivity, and the behavioral deficits of mice at PND 5–7 (Wang et al., 2016). Therefore, in this study, we did experiments on PND7 mice, and after being exposed to propofol for 6 hr, the escape latency was prolonged with the decreased number of crossing platform, which was reversed by the treatment of RTA‐408, indicating that RTA‐408 could strengthen the learning and memory capacity of propofol‐induced mice.

In our study, we found the expression of Nrf2 protein in hippocampus was reduced after the propofol injection. Therefore, we speculated that Nrf2 activation might also play a protective role in the neonatal mice induced by propofol. Previous study found the mice lacking the Nrf2 transcription had increased oxidative stress in hippocampus, thus resulting in serious cognitive impairment, which was further illustrated by a marked amplification of the oxidative stress and inflammatory response factor (McNeilly et al., 2016). Besides, the activation of Nrf2 would improve the cognitive function of PS1V97L‐Tg mice model of AD (Tian et al., 2019). As reported, compounds activating the Nrf2 pathway, which starts the transcription of antioxidant enzymes, such as BDNF (a growth factor that promotes neuronal survival), have been reported to protect the neurons from a variety of injury (Shi et al., 2018). Debolina Ghosh and his groups also revealed Nrf2 activator treatment additively improve redox glutathione levels and neuron survival in aging and in Alzheimer mouse neurons (Ghosh et al., 2013). RTA‐408, as a synthetic triterpenoid compound, is found to be able to potently activate Nrf2, thus promoting antioxidant and anti‐inflammatory effects in neurodegenerative diseases (Shekh‐Ahmad et al., 2018), indicating that RTA‐408 may increase the survival of propofol‐induced hippocampal neurons via activating Nrf2 expression. Furthermore, many studies have reported that Nrf2 can activate a variety of downstream antioxidant genes, like NF‐κB, after being stimulated by inflammation or oxidative stress (Calkins et al., 2009). We also found propofol induced a significant increase of NF‐κB p65 nuclear translocation in the hippocampus of neonatal mice, which was in line with the former researches (Popic et al., 2015). Several researches demonstrated the stimulated nuclear translocation of NF‐κB p65 could be suppressed by RTA‐408 (Sun et al., 2019; Yang et al., 2019). In our study, the hippocampal CA1 neurons of propofol‐induced mice after RTA‐408 treatment were increased, suggesting the enhance survival of neurons by RTA‐408 pretreatment may be related to the activation of Nrf2 and the inhibition of NF‐κB p65 nuclear translocation in propofol‐induced hippocampal of mice. The proinflammatory cytokines mainly including IL‐1, IL‐6, IL‐8, and TNF are small molecular polypeptides synthesized and secreted by immune and nonimmune cells of the body (Dhanisha et al., 2019; Gebremariam et al., 2019), which were shown to be induced in propofol‐induced hippocampus (Milanovic et al., 2016; Yang et al., 2014), being similar as the findings in our study. At present, the anti‐inflammatory medicines can inhibit the activation of NF‐ κB pathway, and antioxidant drugs usually perform cell protection function by activating Nrf2 pathway (Choudhury et al., 2015; Mukherjee et al., 2013). As consistent with previous study, the antioxidase activities of GPx, SOD, and CAT in hippocampus of mice were reduced by propofol, indicating that the oxidative response was serious (Alirezaei et al., 2017). In recent years, a large number of studies at home and abroad have found that RTA 408 has inflammatory and antioxidant effects. For example, in the model of seizure‐like activity in vitro, RTA 408 inhibited the production of active oxygen (Shekh‐Ahmad et al., 2018) and up‐regulated the expression of antioxidant gene in diabetic wounds (Rabbani et al., 2018), so as to play the role of antioxidant stress. In addition, RTA‐408 did attenuate airway inflammation in a murine model of ozone‐induced asthma exacerbation (Zhang et al., 2019) and suppressed proinflammatory cytokine levels in interferon‐γ‐stimulated RAW 264.7 macrophage cells (Probst et al., 2015). From our models, the expression of hippocampal inflammatory factors was significantly reduced in propofol‐induced mice, with the increased level of antioxidant stress after RTA‐408 pretreatment, suggesting that it can significantly improve propofol‐induced hippocampal inflammation and oxidative stress in mice.

Furthermore, the study of neonatal anesthetics, including propofol, concerning the neurotoxicity mainly focuses on apoptosis (Zhu et al., 2018). RTA 408 has been reported to play an antiapoptotic role in several studies. The in vitro experiments showed RTA 408 (10 and 100 nM) treatment can reduce H_2_O_2_‐induced apoptosis of human retinal pigment epithelial cells (Chen et al., 2016), and the mice, underwent unilateral ischemia followed by contralateral nephrectomy, exhibited had the improved renal function and the decreased apoptosis after treated with RTA‐408 in vivo (Han et al., 2017). Meanwhile, caspase‐3 had irreplaceable role in cell apoptosis, as the most important end shear enzyme in the process of apoptosis (Zhou et al., 2018). In our experiment, propofol‐induced apoptosis of hippocampal neurons and expression of caspase‐3 were inhibited with the treatment of RTA 408 with the decreased loss of NeuN (+) and Map2 (+) principal cells, PV(+) interneurons, and CaMKII (+) pyramidal neurons in the hippocampal CA1 areas of mice, indicating that RTA 408 may play a protective role in propofol‐induced cognitive impairment.

To sum up, RTA‐408 can be used to improve learning and memory capacity of propofol‐induced neonatal mice by increasing the survival of neurons, lowering the inflammatory response and oxidative stress in hippocampus, and reducing the apoptosis of neurons, which may be correlated with the activation of Nrf2 and the inhibition of NF‐κB p65 nuclear translocation. However, there exist several limitations: First, we did not measure the actual plasma concentrations of propofol as it is technically difficult to place an intravenous line and obtain blood in PND7 mice; second, the extrahippocampal regions were not examined due to time and funding constraints; finally, further studies in humans will be needed to confirm the ability of RTA‐408 in propofol‐induced cognitive impairment.

## CONFLICT OF INTEREST

None of the authors have any competing interests.

## AUTHOR CONTRIBUTION

Ling Zhang designed and wrote the study. Qian Zhou performed and analyzed the study. Chun‐Li Zhou wrote and revised the study.

## Data Availability

The datasets supporting the conclusions of this article are included within the article.
